# Birth Status, Child Growth, and Adult Outcomes in Low- and Middle-Income Countries^☆^

**DOI:** 10.1016/j.jpeds.2013.08.012

**Published:** 2013-12

**Authors:** Aryeh D. Stein, Fernando C. Barros, Santosh K. Bhargava, Wei Hao, Bernardo L. Horta, Nanette Lee, Christopher W. Kuzawa, Reynaldo Martorell, Siddarth Ramji, Alan Stein, Linda Richter, Consortium of Health-Orientated Research in Transitioning Societies (COHORTS) investigators

**Affiliations:** 1 Hubert Department of Global Health, Rollins School of Public Health, Emory University, Atlanta, GA; 2 MRC/Wits Developmental Pathways for Health Research Unit, Faculty of Health Sciences, University of the Witwatersrand, Johannesburg, South Africa; 3 Postgraduate Program in Health and Behavior, Universidade Católica de Pelotas, Brazil; 4 Department of Pediatrics, S. L. Jain Hospital, Delhi, India; 5 Postgraduate Program in Epidemiology, Universidade Federal de Pelotas, Rio Grande do Sul, Brazil; 6 Office of Population Studies Foundation, University of San Carlos, Cebu, Philippines; 7 Department of Anthropology and Institute for Policy Research, Northwestern University, Evanston, IL; 8 Department of Pediatrics, Maulana Azad Medical College, New Delhi, India; 9 Section of Child and Adolescent Psychiatry, University of Oxford, Oxford, United Kingdom; 10 Human Sciences Research Council, Durban, South Africa

**Keywords:** AGA, Appropriate for gestational age, BMI, Body mass index, GA, Gestational age, IFG, Impaired fasting glucose, LGA, Large for gestational age, LMP, Last menstrual period, SGA, Small for gestational age

## Abstract

**Objective:**

To assess the impact of being born preterm or small for gestational age (SGA) on several adult outcomes.

**Study design:**

We analyzed data for 4518 adult participants in 5 birth cohorts from Brazil, Guatemala, India, the Philippines, and South Africa.

**Results:**

In the study population, 12.8% of males and 11.9% of females were born preterm, and 26.8% of males and 22.4% of females were born term but SGA. Adults born preterm were 1.11 cm shorter (95% CI, 0.57-1.65 cm), and those born term but SGA were 2.35 cm shorter (95% CI, 1.93-2.77 cm) compared with those born at term and appropriate size for gestational age. Blood pressure and blood glucose level did not differ by birth category. Compared with those born term and at appropriate size for gestational age, schooling attainment was 0.44 years lower (95% CI, 0.17-0.71 years) in those born preterm and 0.41 years lower (95% CI, 0.20-0.62 years) in those born term but SGA.

**Conclusion:**

Being born preterm or term but SGA is associated with persistent deficits in adult height and schooling, but is not related to blood pressure or blood glucose level in low- and middle-income settings. Increased postnatal growth is associated with gains in height and schooling regardless of birth status, but not with increases in blood pressure or blood glucose level.

Growth failure in childhood, usually measured as stunting (height for age <−2.0 SDs compared with the reference population), is associated with short stature in adulthood and with lower schooling attainment.^1^ Multiple studies, primarily but not exclusively from high-income countries, have found inverse associations between size at birth and later blood pressure and blood glucose levels^2,3^; however, many of those studies did not pay adequate attention to the potential independent contributions of gestational age (GA) and birth size according to GA. Although both prematurity (ie, birth before 37 completed weeks gestation) and being born small for GA (SGA; typically defined as birth at <10th percentile of birth weight for GA) are associated with increased risk of neonatal mortality,^4^ increased emphasis on the identification and care of such infants has led to a significant decrease in mortality, such that these infants are increasingly surviving to adulthood. Nevertheless, the prevalence of preterm births and SGA births remains high in many populations,^5^ and although preterm birth and SGA status have been associated with undernutrition at age 2 years,^6^ the later growth patterns of children born preterm have not been examined extensively, especially in low- and middle-income countries. Furthermore, whether any potential adverse impact of prematurity or SGA status on later outcomes might be mitigated or potentiated by the pattern of postnatal growth is unclear.

We previously reported that size at birth and growth patterns in childhood are related to attained adult height^7^ and body composition,^8^ schooling,^9^ and blood pressure^10^ and glucose^11^ levels in young adulthood in 5 lower and middle-income countries. Specifically, growth during the first 2 years of life is strongly associated with adult height, but not with elevated blood pressure or glucose levels, and growth later in childhood and through adolescence, especially gains in weight, is associated with increased risk for hypertension and impaired fasting glucose (IFG).^12^ Those analyses did not systematically examine whether the association between child growth patterns and adult outcomes varied among individuals differing by preterm status or by size for GA at birth. Understanding whether postnatal growth patterns affect risk differentially for individuals born preterm or SGA has implications for the management of these infants. Thus, we conducted an analysis of child growth and adult health in 5 low- and middle-income countries to investigate the association of both preterm status and weight for GA with later growth, schooling attainment, and cardiometabolic outcomes.

## Methods

The Consortium of Health-Orientated Research in Transitioning Societies is a collaborative endeavor pooling data from birth cohorts in 5 low- and middle-income countries: Brazil, Guatemala, India, the Philippines, and South Africa.^13^ Descriptions of these cohorts are available elsewhere.^14-18^ All 5 cohorts were established during gestation or at delivery, included at least 1000 individuals under study since birth, had multiple anthropometric measures obtained during childhood, and at the time of establishment of the collaboration had reached at least age 15 years (Table I; available at www.jpeds.com). The youngest cohort (South Africa) has completed data collection at age 18 years; we used those more recent data in the present analysis. All field work was conducted under protocols approved by the respective Ethical Review Committees, and all subjects (or their parents, as appropriate) gave informed consent.

### Measures at Delivery

In India and Guatemala, birth weight was measured by research teams. In the Philippines, birth weight was measured by birth attendants using hanging scales for home births and was obtained from hospital records for hospital births. In Brazil and South Africa, birth weight was measured by birth attendants in hospitals and was extracted from the hospital birth records. In Guatemala, India, and the Philippines, birth length was measured by the research teams using portable length boards within 15 days of delivery. Birth length was not measured in Brazil or South Africa.

GA was calculated based on the reported date of the last menstrual period (LMP) and the date of delivery. In Guatemala and India, ongoing surveillance was used to identify incident pregnancies. In Brazil and South Africa, the date of LMP was extracted from the medical records. In the Philippines, the date of LMP was reported by the mother at the time of recruitment; the Ballard score, based on clinical assessment of the newborn's neuromuscular and physical characteristics,^19^ was used for infants with low birth weight. The Ballard score was used in the Philippines because in 1982, it was considered more accurate than LMP, especially in populations in which a significant proportion of women did not experience a menstrual period between pregnancies. The Ballard score was used to define GA whenever available.

We classified subjects born at <259 days post-LMP (37 completed weeks of gestation) as preterm, and those born at ≥294 or more days post-LMP (42 completed weeks) as postterm. We classified term and postterm infants as SGA if they were below the 10th percentile of the sex-specific birth weight for GA distribution,^20^ as large for GA (LGA) if they were above the 90th percentile, or as appropriate for GA (AGA). There were insufficient sample sizes within individual cohorts to permit classification of preterm infants by SGA status.

### Measures in Childhood

Each of the 5 study cohorts collected anthropometric measures (height and weight) at study-specific intervals.^14-18^ Across the 5 cohorts, common ages at measurement included 12 months for subsamples in Brazil and South Africa, 24 months, and an age that for convenience we designate as mid-childhood (48 months for the cohorts from Brazil, Guatemala, and India; 60 months for the South African cohort; and 102 months for the Philippine cohort). We computed height-for-age *z*-scores using the current World Health Organization reference population data.^21^

### Measures in Adulthood

In all 5 cohorts, standing height was measured using a fixed stadiometer and weight was measured with a portable scale. Blood pressure was measured using mercury sphygmomanometers in the Philippines and digital devices in the other cohorts (Omron HEM-629 in Brazil [Omron Healthcare Inc, Lake Forest, Illinois], A&D Medical UA-767 in Guatemala [A&D Medical, Milpitas, California]; Omron M6 in South Africa; Omron 711 in India). Appropriate cuff sizes were used, and measurements were made with the subjects seated after a 5- to 10-minute rest. Field protocols differed across the cohorts; for Brazil, India, and South Africa, the mean of 2 measurements (for South Africa, 3 measurements were taken, but the first was discarded) was used; for the Philippines and Guatemala, 3 measures were averaged. In all cohorts but Brazil, the research team collected fasting blood samples to determine glucose levels; in Brazil, random blood samples were obtained, and values were adjusted for the time since the last meal.^22^ In the Philippines, glucose levels were assayed from whole venous blood samples. Because glucometers overestimate glucose concentrations in whole venous blood compared with standard laboratory methods,^23^ we subtracted 0.97 mmol/L from the values in the Philippines cohort to estimate the best equivalent to venous plasma as analyzed by a laboratory autoanalyzer.^24^ The highest grade of schooling completed was ascertained by questionnaire.

Body mass index (BMI) was calculated as weight in kilograms divided by height squared in meters. Prehypertension or hypertension was defined as systolic blood pressure ≥120 mm Hg or diastolic blood pressure ≥70 mm Hg, or the use of antihypertensive medication (reported by <0.5% of participants).^25^ Prehypertension was included in our outcome because of the young age of the study participants. IFG was defined as blood glucose ≥6.1 mmol/L and <7.0 mmol/L, and diabetes was defined as blood glucose concentration ≥7.0 mmol/L or a reported previous medical diagnosis of diabetes.^26^ IFG and diabetes were combined for analysis. Schooling was classified as completion of secondary school (based on site-specific criteria: completion of 12th grade in Brazil, India, and South Africa; 11th grade in Guatemala; and 10th grade in the Philippines) or not.

### Statistical Analyses

Our study population comprised the 4518 individuals (21.7% of the initial birth cohorts) for whom data were available for sex, GA and weight at birth, length at age 12 and 24 months and in mid-childhood, and adult height. Study exclusions are summarized in Table II (available at www.jpeds.com). For analyses focusing on weight or BMI, we excluded 5 individuals with missing data; for blood pressure, we excluded 110 individuals with missing data or who were pregnant; for glucose, we excluded 772 individuals with missing data or who were pregnant; and for schooling, we excluded 65 individuals with missing data.

We computed descriptive statistics by site for the key exposure and outcome variables. We compared adult height, BMI, blood pressure, glucose level, and schooling attainment among groups using ANOVA, and compared prevalences using categorical approaches. Specifically, we compared outcomes among 4 birth categories: those born preterm, those born term-SGA, those born term-AGA, and those born term-LGA. The latter 3 groups also included those born postterm.

We assessed patterns of growth from birth to adulthood by computing the changes in length within each period of childhood, and compared these data across the 3 birth categories. To assess whether the patterns of childhood growth were differentially related to adult measures across birth status categories, we used conditional length measures to control for the tendency of growth to track over time.^27,28^ We computed these conditional lengths as the residuals from site- and sex-specific linear regression models in which the dependent variable was length at any given age and the predictor variables were birth weight and any previously recorded lengths. We anchored the models on birth weight because birth length data were not available for 2 of the cohorts; in the 3 cohorts with available birth length and birth weight, the results were very similar using either anchor. The residual thus obtained may be interpreted as the deviation from the child's predicted growth trajectory, and hence is a measure of relatively accelerated or retarded growth within an age interval. Conditional length at any age is, by definition, uncorrelated with birth weight or conditional length at any other age.

Because site- and sex-stratified estimates were similar, we conducted site- and sex-pooled analyses. We compared models for fasting glucose and IFG with and without adjustment for adult BMI; there were no meaningful differences, and thus only the unadjusted models are presented. All analyses were performed with SAS version 9.3 (SAS Institute, Cary, North Carolina).

## Results

Overall, 12.8% of males and 11.9% of females were born preterm, 26.8% of males and 22.4% of females were born term-SGA, and 2.1% of males and 2.0% of females were born term-LGA (Table III). The prevalences of preterm births, SGA and LGA status, and all adult outcomes differed across the cohorts (Table IV; available at www.jpeds.com). There were only small differences in period-specific growth increments across birth status categories (Table V). Boys grew more than girls in the period from mid-childhood to adulthood.

### Adult Outcomes in Relation to GA and GA-Adjusted Size at Delivery

Compared with adults born term-AGA, those born preterm were 1.11 cm shorter (95% CI, 0.57-1.65 cm), those born term-SGA were 2.35 cm shorter (95% CI, 1.93-2.77 cm) and those born term-LGA were 2.88 cm taller (95% CI, 1.65-4.12 cm) (Table VI). Adults born preterm were 0.29 kg/m^2^ (95% CI, −0.07 to 0.65 kg/m^2^) thinner, those born term-SGA were 0.78 kg/m^2^ (95% CI, 0.49-1.06 kg/m^2^) thinner, and those born term-LGA had BMI values 0.56 kg/m^2^ (95% CI, −0.27 to 1.38 kg/m^2^) higher than those born term-AGA. Blood pressure and glucose did not differ significantly by birth category. Schooling attainment was lower in those born preterm and term-SGA compared with those born term-AGA. Patterns for prehypertension/hypertension, for IFG/diabetes, and for completion of secondary school were consistent with the associations for the continuous variables. Further adjustment for adult height in models for blood pressure and prehypertension/hypertension did not change the estimates substantively. There was no evidence of heterogeneity of estimates across the cohorts, except those for schooling (Table VII; available at www.jpeds.com).

### Adult Outcomes in Relation to Postnatal Growth

Birth weight and conditional length at 12 months, 24 months, and mid-childhood were each positively associated with adult height, with similar estimates across birth categories, except that the coefficient for conditional length at 12 months was stronger for the preterm group compared with the other groups (*P* < .05; Table VIII). For systolic blood pressure, there were associations with birth weight that were heterogenous across birth categories, as well as positive associations with conditional size at all later periods. For diastolic blood pressure, the coefficient for birth weight differed across groups, being strongest for those born preterm (*P* < .05). Fasting glucose was not significantly associated with birth weight or conditional lengths in any group. Birth weight was more strongly associated with schooling among those born term-SGA than the other birth categories (*P* < .05). Postnatal growth was associated with schooling attainment in all 3 birth categories, with no evidence of heterogeneity. Patterns of association for the binary variables were consistent with those derived from the continuous measures.

## Discussion

We have described patterns of association relating status at birth and postnatal growth patterns simultaneously to several important adult outcomes in 5 prospective birth cohorts in low- and middle-income countries. Both preterm birth and term-SGA birth were associated with adult shortness, thinness, and reduced school attainment, but little difference by birth status in adult blood pressure or glucose levels. Our core dataset included more than 4500 individuals, although not all provided data for all outcomes. The 5 cohorts represent a range of socioeconomic and political backgrounds. We observed little heterogeneity in postnatal growth patterns by birth status. These results extend our previous work^7-12^ by considering potential heterogeneity in the effects of postnatal growth by birth status and by including new data from the South African cohort at age 18 years. Our key finding of very little heterogeneity across the 5 cohorts in any of the estimates of association despite large differences among the cohorts in the prevalence of preterm and term-SGA births and the adult outcomes reinforces the robustness of these findings.

Country-level data for prematurity are not available for most low- and middle-income countires. Recent estimates of the prevalence of preterm births are 16.5% for Bangladesh, 12.3% for Gambia, and 23.1% for Nepal, compared with ∼15% in Brazil and ∼6.0% in the United Kingdom and Sweden,^29^ and prevalence appears to be rising globally.^4^ Our results are somewhat lower than these estimates from other low- and middle-income countries; this may reflect differences in the method of ascertainment of GA across the studies, or underlying differences between our study cohorts and other nationally representative samples.

In high-income countries, most preterm infants catch up with term infants in weight and height by age 12-24 months,^30^ although very preterm infants weighing <1.5 kg still demonstrate deficits in late childhood.^31^ The available evidence suggests that low birth weight and preterm birth are risk factors for undernutrition in young children in low- and middle-income countries.^6^ Our data extend those findings to adulthood.

Our results are important because preterm births and fetal and postnatal growth restriction are common; indeed, the conflation of preterm births and SGA births may be greater in low- and middle-income countries compared with high-income countries.^5,29,32,33^ It is well established that fetal growth restriction is associated with development of cardiometabolic disease,^3^ and that growth restriction at age 2 years is associated with adult shortness^7^ and reduced cognitive functioning,^1^ both of which are important measures of human capital. Critically, our findings suggest that the deficits are established before delivery, because both preterm and term-SGA status are associated with adult short stature and lower levels of schooling.

We were able to include data for 21.7% of the original birth cohorts in our analysis. The primary reasons for attrition were a lack of data on adult height (for >50% of the birth cohort, mostly from India and reflecting systematic population relocations) and missing data at 12 months (for 18% of the birth cohort, mostly from Brazil and South Africa and reflecting systematic sampling at that age). Because the reasons for attrition are not related to individual characteristics, we believe that our estimates are unlikely to be seriously biased. Because birth length was not recorded for Brazil or South Africa and was missing for some of the Guatemalan and Indian samples, we used birth weight to anchor our growth models. Birth length and birth weight correlated at 0.7, and for the 3 sites with both birth length and birth weight (Guatemala, India, and the Philippines), the results were very similar regardless of the birth measure used. GA was estimated from the date of LMP, and ultrasound dating was not available in any of the communities at the time of field work. However, in Guatemala and India there was active surveillance of incident pregnancies, so misclassification of LMP is unlikely to have been large, and in the Philippines all low birth weight infants were examined using Ballard criteria to differentiate preterm births from term-SGA births. Ballard scores may overestimate GA in preterm infants compared with ultrasonography.^34^ Residual misclassification would serve to reduce the study's power to detect between-group differences. In low- and middle-income countries, up to one-half of all low birth weight infants are preterm.^35^ Despite our large pooled dataset, we were unable to differentiate preterm-SGA from preterm-AGA infants, particularly for within-site analyses because of small cell sizes. These 2 groups are likely to have heterogeneous postnatal pathways and adult outcomes.

Recent decades have brought major advances in the ability to ensure survival of preterm infants,^4^ but most of these benefits have accrued in high-income countries. Two-thirds of the preterm births in our dataset were late preterm, reflecting the poor survival of early preterm infants in these communities. Thus, our results might not generalize to the experience of early preterm births.

Despite encouraging long-term reductions in the prevalence of stunting,^36^ postnatal growth failure remains widely prevalent in low- and middle-income countries.^32^ Growth failure is associated with important aspects of adult human capital,^1^ and evidence from our Guatemalan cohort suggests that improved early-life nutrition reduces the prevalence of severe stunting^37^ and improves cognitive functioning^38^ and economic productivity.^39^ Our results suggest that these findings should hold true regardless of birth status, and reinforce the fact that enhancing linear growth will not adversely impact blood pressure or glucose levels. Given the fact that rapid increases in weight for length adversely affect cardiometabolic risk factors,^12^ the challenge lies in how to improve childhood linear growth without increasing weight for length.

We conclude that preterm or term-SGA birth is associated with shorter adult height and reduced schooling attainment in low- and middle-income countries. Increased postnatal growth is associated with gains in these outcomes, but not with increases in blood pressure or glucose level, regardless of birth status. These results are encouraging for programs seeking to improve child nutrition in the first 1000 days of life.

## Figures and Tables

**Table I:** Overview of the 5 cohorts providing data for these analyses

Cohort name	Design	Enrollment year	Cohort description	Most recent follow-up, year
INCAP Nutrition Trial Cohort Study, Guatemala	Community intervention trial	1969-77	Intervention trial of high-energy and protein supplementation in women and children aged <7 y in 1969 and born during 1969-1977 in 4 villages	2003-2005
New Delhi Birth Cohort Study, India	Prospective cohort	1969-72	Births to a population of married women living in a defined area of Delhi, a primarily middle-class sample	2006-2009
Cebu Longitudinal Health and Nutrition Survey, Philippines	Prospective cohort	1983-4	Pregnant women living in 33 randomly selected neighborhoods; 75% urban, all social classes	2005
Birth-to-20 Study, South Africa	Prospective cohort	1990	Babies born to pregnant women living in a defined urban area; a primarily poor, black sample	2008
Pelotas 1982 Birth Cohort Study	Prospective cohort	1982	99% of all births in city's maternity hospital in 1982; all social classes	2005

*INCAP*, Institute of Nutrition of Central America and Panama.

**Table II:** Losses to follow-up and exclusions from analysis

	Brazil	Guatemala	India	Philippines	South Africa	All sites
Total number of births	5913	1041	7530	3080	3273	20 837
Missing adult height (not followed up)	1727	456	6006	1043	1338	10 570
Missing GA (unable to compute preterm status)	823	135	146	16	39	1159
Missing birth weight (unable to compute size for GA status)	1	11	73	31	3	119
Missing length at 12 mo (unable to estimate growth in first year of life)	2463	27	244	116	974	3824
Missing length at 24 mo (unable to compute growth in second year of life)	54	26	55	33	243	411
Missing length at mid-childhood (unable to compute growth in mid-childhood)	41	107	10	13	65	236
Subjects included in the study	804	279	996	1828	611	4518

**Table IV:** Selected characteristics at birth and follow-up among 4518 participants in 5 birth cohorts in lower- and middle-income countries, by site

Characteristic	Brazil		Guatemala		India		Philippines		South Africa	
Male	Female	Male	Female	Male	Female	Male	Female	Male	Female
Status at delivery, %	n = 397	n = 407	n = 149	n = 130	n = 572	n = 424	n = 971	n = 857	n = 285	n = 326
Preterm (<37 completed weeks)∗	4.5	5.9	15.4	7.7	15.2	11.8	14.6	14.7	11.6	13.5
Not preterm (37-42 completed weeks)	87.2	79.1	72.5	73.1	75.9	74.8	78.7	76.7	87.4	86.2
Postterm (≥42 completed weeks)	8.3	15.0	12.1	19.2	8.9	13.4	6.7	8.6	1.1	0.3
Not preterm-SGA†	15.6	14.0	31.5	23.1	42.5	41.0	25.2	20.7	14.0	13.2
Not preterm-LGA‡	5.3	4.7	3.4	2.3	0.9	0.5	0.7	1.4	4.2	1.8
Height, cm, mean ± SD	173.8 ± 6.4	161.6 ± 5.7	162.8 ± 6.2	151.3 ± 5.2	169.4 ± 6.2	155.2 ± 5.6	163.0 ± 5.9	151.0 ± 5.4	171.2 ± 7.3	159.7 ± 6.4
	n = 397	n = 407	n = 149	n = 130	n = 572	n = 424	n = 971	n = 852	n = 285	n = 326
Weight, kg, mean ± SD	73.8 ± 15.0	61.3 ± 12.3	64.0 ± 10.1	61.5 ± 11.2	71.9 ± 14.0	60.0 ± 13.3	56.0 ± 9.3	46.3 ± 8.0	60.6, 12.2	58.2 ± 12.0
BMI, mean ± SD	24.4 ± 4.5	23.5 ± 4.5	24.1 ± 3.3	26.8 ± 4.5	25.0 ± 4.3	24.8 ± 5.0	21.0 ± 3.1	20.3 ± 3.1	20.7 ± 3.7	22.8 ± 4.4
Blood pressure, mmHg, mean ± SD	n = 396	n = 407	n = 140	n = 124	n = 567	n = 418	n = 971	n = 790	n = 277	n = 318
Systolic blood pressure	123.5 ± 14.2	111.9 ± 12.3	116.6 ± 9.7	108.6 ± 11.1	117.9 ± 11.2	107.0 ± 10.8	111.8 ± 10.9	99.6 ± 10.0	119.8 ± 10.7	114.1 ± 9.9
Diastolic blood pressure	75.7 ± 11.2	72.3 ± 10.3	72.1 ± 9.3	70.1 ± 8.8	77.7 ± 10.2	73.7 ± 9.1	75.9 ± 9.6	68.2 ± 8.6	71.0 ± 9.0	71.7 ± 8.6
Prehypertension/hypertension, %	41.7	22.1	22.1	13.7	41.1	25.6	44.2	11.9	21.7	14.5
Glucose metabolism	n = 339	n = 359	n = 98	n = 107	n = 559	n = 413	n = 846	n = 683	n = 167	n = 175
Fasting glucose, mmol/L, mean ± SD	5.2 ± 0.7	4.9 ± 0.7	5.2 ± 0.6	5.1 ± 1.2	5.5 ± 1.1	5.4 ± 0.8	4.7 ± 0.6	4.6 ± 0.5	4.6 ± 0.5	4.5 ± 0.4
IFG/diabetes, %	10.9	5.6	4.1	2.8	21.5	15.3	0.9	1.0	0.0	0.0
Schooling	n = 369	n = 387	n = 146	n = 129	n = 572	n = 424	n = 971	n = 857	n = 277	n = 321
Highest grade attained, y, mean ± SD	9.5 ± 3.0	10.3 ± 2.9	5.1 ± 3.5	4.9 ± 3.6	13.3 ± 3.4	14.2 ± 2.6	10.4 ± 3.3	11.5 ± 2.8	10.8 ± 1.5	11.3 ± 1.3
Completed secondary school, %§	14.4	22.2	11.6	9.3	85.7	94.6	68.3	85.4	47.3	70.7

∗ Preterm births are not further categorized as SGA or AGA owing to limited sample size.

† SGA: below the 10th percentile of sex-specific birth weight for GA.

‡ LGA: above the 90th percentile of sex-specific birth weight for GA.

§ 12 years for Brazil, India, and South Africa; 11 years for Guatemala; and 10 years for the Philippines.

**Table VII:** Differences in adult height, blood pressure, fasting glucose level, and schooling attainment in relation to preterm status and size at delivery among participants in 5 birth cohorts in lower- and middle-income countries, by site

	Brazil		Guatemala		India		Philippines		South Africa		*P* value∗
Coefficient	95% CI	Coefficient	95% CI	Coefficient	95% CI	Coefficient	95% CI	Coefficient	95% CI
Height, cm											
Preterm	−0.41	−2.25 to 1.44	−1.51	−3.66 to 0.64	−0.87	−1.99 to 0.26	−1.18	−1.93 to −0.43	−1.55	−3.20 to 0.09	.20
Not preterm-SGA†	−2.97	−4.13 to −1.81	−2.77	−4.32 to −1.22	−2.07	−2.86 to −1.28	−2.00	−2.63 to −1.37	−3.37	−4.97 to −1.78	
Not preterm-LGA‡	3.94	2.05 to 5.83	3.41	−0.62 to 7.43	6.59	2.23 to 10.96	1.75	−0.79 to 4.29	-0.07	−3.28 to 3.13	
Weight, kg											
Preterm	−0.42	−4.68 to 3.83	0.63	−3.36 to 4.63	-4.19	−6.77 to −1.61	−0.52	−1.68 to 0.64	−2.05	−4.95 to 0.85	.23
Not preterm-SGA	−4.73	−7.40 to −2.05	-4.18	−7.07 to −1.29	−4.42	−6.22 to −2.61	−2.67	−3.64 to −1.70	−4.65	−7.46 to −1.84	
Not preterm-LGA	4.47	0.12 to 8.82	3.60	−3.89 to 11.08	10.17	0.16 to 20.19	1.99	−1.94 to 5.91	4.63	−1.04 to 10.29	
BMI											
Preterm	0.00	−1.42 to 1.42	0.55	−0.92 to 2.02	−1.27	−2.15 to −0.39	0.07	−0.35 to 0.48	-0.28	−1.28 to 0.71	.41
Not preterm-SGA	−0.78	−1.67 to 0.11	−0.94	−2.00 to 0.12	−1.04	−1.65 to −0.42	−0.60	−0.94 to −0.25	−0.85	−1.81 to 0.12	
Not preterm-LGA	0.42	−1.03 to 1.87	0.32	−2.43 to 3.07	1.74	−1.68 to 5.16	0.29	−1.11 to 1.69	1.63	−0.31 to 3.57	
Systolic blood pressure, mmHg											
Preterm	−0.71	−4.86 to 3.45	0.47	−3.56 to 4.50	-0.86	−3.01 to 1.28	0.04	−1.40 to 1.49	−1.56	−4.12 to 1.00	1.00
Not preterm-SGA	−0.38	−2.99 to 2.23	−0.83	−3.79 to 2.13	0.24	−1.26 to 1.74	0.24	−0.96 to 1.44	0.06	−2.39 to 2.51	
Not preterm-LGA	2.51	−1.74 to 6.76	0.56	−6.88 to 8.00	2.08	−6.21 to 10.37	2.74	−2.04 to 7.51	1.76	−3.12 to 6.64	
Diastolic blood pressure, mmHg											
Preterm	−0.69	−4.06 to 2.68	0.65	−2.85 to 4.15	-0.47	−2.36 to 1.42	0.16	−1.09 to 1.41	−0.95	−3.13 to 1.24	.96
Not preterm-SGA	−1.35	−3.47 to 0.77	−0.29	−2.86 to 2.28	0.16	−1.16 to 1.48	−0.54	−1.58 to 0.50	0.10	−1.99 to 2.19	
Not preterm-LGA	−1.18	−4.62 to 2.27	4.59	−1.88 to 11.05	0.16	−7.13 to 7.45	0.80	−3.33 to 4.93	0.08	−4.08 to 4.23	
Fasting glucose, mmol/L											
Preterm	0.07	−0.16 to 0.30	0.31	−0.10 to 0.73	−0.01	−0.20 to 0.18	0.08	0.00 to 0.16	0.02	−0.12 to 0.15	.68
Not preterm-SGA	0.02	−0.12 to 0.17	−0.04	−0.35 to 0.28	0.08	−0.05 to 0.21	−0.01	−0.08 to 0.05	0.06	−0.07 to 0.19	
Not preterm-LGA	−0.02	−0.27 to 0.22	0.06	−0.68 to 0.80	−0.24	−0.96 to 0.48	−0.10	−0.35 to 0.14	0.08	−0.16 to 0.31	
Completed years of schooling											
Preterm	−0.06	−1.01 to 0.89	0.88	−0.47 to 2.24	−1.14	−1.73 to −0.56	−0.43	−0.84 to −0.01	−0.05	−0.39 to 0.28	<.01
Not preterm-SGA	−1.34	−1.93 to -0.74	0.76	−0.22 to 1.75	−0.58	−0.99 to −0.17	−0.28	−0.63 to 0.06	−0.39	−0.71 to −0.06	
Not preterm-LGA	0.86	−0.10 to 1.82	1.24	−1.30 to 3.78	0.91	−1.36 to 3.18	−0.46	−1.87 to 0.94	−0.16	−0.82 to 0.51	
Prehypertension/hypertension, OR											
Preterm	0.89	0.44 to 1.81	0.42	0.12 to 1.51	0.76	0.50 to 1.17	0.85	0.61 to 1.18	1.19	0.63 to 2.27	.96
Not preterm-SGA	0.93	0.60 to 1.44	1.17	0.58 to 2.38	0.99	0.74 to 1.32	0.99	0.76 to 1.29	1.31	0.72 to 2.38	
Not preterm-LGA	0.99	0.49 to 1.98	1.34	0.25 to 7.22	1.28	0.28 to 5.94	1.06	0.35 to 3.24	1.67	0.57 to 4.88	
IFG/diabetes, OR											
Preterm	1.42	0.48 to 4.23	4.75	0.87 to 25.81	1.02	0.62 to 1.68	2.61	0.76 to 9.02	(-)§		.95
Not preterm-SGA	1.06	0.50 to 2.26	0.64	0.06 to 6.39	1.00	0.70 to 1.42	1.51	0.44 to 5.19	(-)		
Not preterm-LGA¶											
Completed secondary school, OR											
Preterm	0.70	0.28 to 1.72	1.25	0.38 to 4.10	0.36	0.20 to 0.66	0.77	0.56 to 1.05	0.85	0.51 to 1.42	.05
Not preterm-SGA	0.27	0.12 to 0.59	1.24	0.51 to 3.02	0.50	0.31 to 0.80	0.79	0.61 to 1.03	0.62	0.38 to 1.01	
Not preterm-LGA	1.90	0.93 to 3.91	(-)§		(-)§		0.48	0.17 to 1.31	1.02	0.37 to 2.82	

Separate models were run for each adult variable. Estimates are derived from linear (height, weight, BMI, blood pressure, fasting glucose level, schooling attainment) or logistic (prehypertension/hypertension, IFG/diabetes, completion of secondary school) regression models, respectively, and are adjusted for sex and age at adult assessment. Reference category is Term (≥37 completed weeks gestation) and AGA. Site-pooled models are also adjusted for site.

∗ Test for heterogeneity of estimates across sites (Wald test; 15 df unless individual cohort/birth status strata could not be analyzed).

† SGA: below the 10th percentile for sex-specific weight for GA.

‡ LGA: above the 90th percentile for sex-specific weight for GA.

§ Insufficient sample size to derive stable estimates. Model fits for the Guatemala and Philippines data are questionable.

¶ Only 3 participants across the 5 sites who were Term-LGA had prediabetes/diabetes; not analyzed further.

**Table III:** Selected characteristics at birth and follow-up of 4518 participants in 5 birth cohorts in low- and middle-income countries

	Males (n = 2374)	Females (n = 2144)
Status at delivery, %		
Preterm (<37 completed weeks)∗	12.8	11.9
Term (37-42 completed weeks)	80.1	78.0
Postterm (>42 completed weeks)	7.2	10.2
Not preterm-SGA†	26.8	22.4
Not preterm-LGA‡	2.1	2.0
Height, cm, mean ± SD	167.3 ± 7.6	155.2 ± 7.1
Weight, kg, mean ± SD§	63.9 ± 14.3	54.6 ± 12.8
BMI, mean ± SD	22.7 ± 4.2	22.6 ± 4.6
Blood pressure, mmHg, mean ± SD¶		
Systolic blood pressure	116.5 ± 12.3	106.3 ± 12.2
Diastolic blood pressure	75.5 ± 10.2	70.8 ± 9.3
Prehypertension/hypertension, %	39.0	17.2
Glucose metabolism∗∗		
Fasting glucose, mmol/L, mean ± SD	5.0 ± 0.8	4.9 ± 0.8
IFG/diabetes, %	8.4	5.4
Schooling††		
Highest grade attained, y, mean ± SD	10.7 ± 3.7	11.4 ± 3.4
Completed secondary school, %	58.0	68.8

∗ Preterm births are not further categorized as SGA or AGA owing to limitations of sample size.

† SGA: below the 10th percentile of sex-specific birth weight for GA.

‡ LGA: above the 90th percentile of sex-specific birth weight for GA.

§ Males, n = 2374; females, n = 2139.

¶ Males, n = 2351; females, n = 2057.

∗∗ Males, n = 2009; females, n = 1737.

†† Males, n = 2335; females, n = 2118.

**Table V:** Increments in length during periods of childhood according to gestational status and size at birth among 4518 participants in birth cohort studies from 5 low- and middle-income countries, by site

	Males				Females			
Not preterm-AGA	Not preterm-LGA∗	Not preterm-SGA†	Preterm	Not preterm-AGA	Not preterm-LGA∗	Not preterm-SGA†	Preterm
Brazil, cm, mean ± SD	n = 296	n = 21	n = 62	n = 18	n = 307	n = 19	n = 57	n = 24
12-24 mo	11.5 ± 2.4	11.8 ± 2.2	11.3 ± 2.5	12.1 ± 2.5	11.8 ± 2.7	11.3 ± 2.5	11.6 ± 2.0	11.6 ± 2.7
24-48 mo	15.4 ± 2.5	15.6 ± 2.1	15.1 ± 2.6	16.6 ± 2.7	15.6 ± 3.0	15.7 ± 3.4	15.5 ± 2.6	15.8 ± 2.9
48 mo-adult	73.1 ± 4.7	75.4 ± 3.7	72.2 ± 5.1	73.2 ± 4.5	61.7 ± 4.4	61.7 ± 4.2	61.4 ± 4.5	61.8 ± 3.4
Guatemala, cm, mean ± SD	n = 74	n = 5	n = 47	n = 23	n = 87	n = 3	n = 30	n = 10
Birth-12 mo	19.5 ± 2.7	17.7 ± 1.7	19.4 ± 2.6	18.3 ± 3.6	19.1 ± 2.1	20.0 ± 2.2	19.5 ± 3.1	19.7 ± 2.7
12-24 mo	8.8 ± 1.9	6.9 ± 2.3	8.1 ± 2.2	8.9 ± 1.7	9.0 ± 2.1	9.0 ± 1.1	9.5 ± 2.1	8.5 ± 1.7
24-48 mo	15.3 ± 1.7	17.0 ± 3.6	15.8 ± 3.5	15.5 ± 2.1	15.4 ± 2.1	15.8 ± 0.7	16.0 ± 2.2	15.8 ± 2.2
48 mo-adult	70.4 ± 4.6	73.1 ± 5.3	68.0 ± 4.4	69.1 ± 4.2	59.1 ± 4.2	58.0 ± 3.6	57.6 ± 4.6	59.8 ± 3.6
India, cm, mean ± SD	n = 237	n = 5	n = 243	n = 87	n = 198	n = 2	n = 174	n = 50
Birth-12 mo	23.0 ± 2.4	22.7 ± 2.1	23.8 ± 2.7	23.4 ± 2.7	22.0 ± 2.6	19.3 ± 4.4	22.2 ± 2.7	22.2 ± 3.4
12-24 mo	9.3 ± 2.0	10.6 ± 3.0	9.0 ± 2.1	9.3 ± 1.9	9.4 ± 1.7	9.4 ± 1.0	9.6 ± 1.9	9.3 ± 1.9
24-48 mo	14.2 ± 2.2	14.7 ± 1.8	14.2 ± 2.2	14.8 ± 2.3	14.5 ± 2.2	17.4 ± 2.0	14.1 ± 2.4	14.7 ± 2.3
48 mo-adult	74.1 ± 4.2	75.9 ± 3.6	73.7 ± 5.0	74.2 ± 5.3	61.2 ± 4.2	64.4 ± 1.2	61.0 ± 4.1	61.1 ± 4.2
Philippines, cm, mean ± SD	n = 577	n = 7	n = 245	n = 142	n = 542	n = 12	n = 177	n = 126
Birth-12 mo	22.0 ± 2.4	21.9 ± 1.5	22.4 ± 2.7	22.4 ± 2.8	21.0 ± 2.5	20.4 ± 2.9	21.3 ± 2.6	21.4 ± 3.0
12-24 mo	8.4 ± 2.1	7.3 ± 2.2	8.7 ± 2.1	8.5 ± 2.0	8.5 ± 2.1	7.7 ± 2.4	8.6 ± 2.2	8.2 ± 2.4
24-102 mo	37.8 ± 3.7	37.9 ± 2.8	37.5 ± 4.0	38.1 ± 4.1	39.3 ± 4.0	40.0 ± 3.4	38.8 ± 4.3	39.1 ± 3.7
102 mo-adult	45.5 ± 3.9	48.1 ± 2.7	45.0 ± 4.0	44.7 ± 3.9	33.5 ± 4.0	34.3 ± 3.1	33.1 ± 4.1	34.0 ± 3.5
South Africa, cm, mean ± SD	n = 200	n = 12	n = 40	n = 33	n = 233	n = 6	n = 43	n = 44
12-24 mo	9.0 ± 2.7	10.2 ± 2.1	9.1 ± 3.4	9.5 ± 2.3	9.9 ± 2.8	8.5 ± 2.8	9.5 ± 3.6	10.3 ± 2.4
24-60 mo	24.1 ± 3.0	23.8 ± 4.1	23.4 ± 3.5	24.5 ± 4.0	24.2 ± 3.6	25.6 ± 6.1	23.7 ± 3.8	24.4 ± 3.1
60 mo-adult	63.9 ± 5.9	61.3 ± 4.3	63.2 ± 4.8	62.2 ± 4.9	52.7 ± 4.6	53.5 ± 7.0	52.0 ± 3.9	52.6 ± 4.8

Preterm births were not further differentiated as to SGA status owing to limitations of sample size. Length at birth not available for cohorts from Brazil and South Africa, and missing for 40 males and 28 females in Guatemala and for 9 males and 2 females in India.

∗ LGA: above the 90th percentile of sex-specific birth weight for GA.

† SGA: below the 10th percentile of sex-specific birth weight for GA.

**Table VI:** Differences in adult height, blood pressure, fasting glucose level, and schooling in relation to gestational status and size at delivery

	Preterm (n = 557)∗		Not preterm-SGA (n = 1118)†		Not preterm-LGA (n = 92)‡	
Coefficient	95% CI	Coefficient	95% CI	Coefficient	95% CI
Height, cm	−1.11	−1.65 to −0.57	−2.35	−2.77 to −1.93	2.88	1.65 to 4.12
Weight, kg§	−1.54	−2.60 to −0.48	−3.71	−4.54 to −2.89	4.00	1.60 to 6.39
BMI§	−0.29	−0.65 to 0.08	−0.78	−1.06 to −0.49	0.56	−0.27 to 1.38
Systolic blood pressure, mmHg§	−0.46	−1.51 to 0.59	0.12	−0.70 to 0.93	2.14	−0.20 to 4.48
Diastolic blood pressur, mmHg§	−0.23	−1.13 to 0.68	−0.30	−1.00 to 0.40	−0.11	−2.13 to 1.90
Fasting glucose, mmol/L§	0.06	−0.02 to 0.13	0.03	−0.03 to 0.08	−0.04	−0.21 to 0.12
Completed years of schooling§	−0.44	−0.71 to −0.17	−0.41	−0.62 to −0.20	0.46	−0.16 to 1.08
Prehypertension/hypertension, OR§	0.84	0.67 to 1.04	1.03	0.87 to 1.21	1.12	0.70 to 1.79
IFG/diabetes, OR§	1.27	0.84 to 1.92	1.04	0.77 to 1.41	(-)¶	
Completed secondary school, OR§^,^∗∗	0.71	0.56 to 0.89	0.68	0.56 to 0.82	1.28	0.75 to 2.18

Estimates are derived from linear (height, weight, BMI, blood pressure, fasting glucose level, and completed years of schooling) or logistic (prehypertension/hypertension, IFG/diabetes, and completion of secondary school) regression models, respectively, and are adjusted for site, sex, and age at adult assessment. *P* values for heterogeneity across sites is <.05 for completed years of schooling and for completion of 12th grade and >.25 for all other measures, using the Wald test (see Table VII for site-specific estimates). The reference category is Term-AGA (neither SGA nor LGA); n = 2751.

∗ <37 completed weeks gestation.

† SGA: below the 10th percentile for sex-specific weight for GA.

‡ LGA: above the 90th percentile for sex-specific weight for GA.

§ Five individuals were excluded from analyses of weight and BMI, 110 were excluded from analyses of blood pressure, 772 were excluded from analyses of glucose, and 65 were excluded from analyses of schooling.

¶ Only 3 participants were not-preterm LGA with diabetes.

∗∗ 12th grade in Brazil, India, and South Africa; 11th grade in Guatemala; and 10th grade in the Philippines.

**Table VIII:** Associations between growth in childhood and adult height, blood pressure, fasting glucose level, and schooling attainment among participants in 5 birth cohorts in low- and middle-income countries, by categories of gestational status and weight at delivery

	Not preterm-AGA		Not preterm-LGA∗		Not preterm-SGA†		Preterm‡	
Coefficient	95% CI	Coefficient	95% CI	Coefficient	95% CI	Coefficient	95% CI
Adult height, cm								
Birth weight	1.59^a^	1.36 to 1.82	2.18^b^	−0.16 to 4.52	1.61^a^	1.13 to 2.10	1.64^a^	1.30 to 1.97
Conditional length at 12 mo	3.21^b^	3.05 to 3.37	3.07	2.05 to 4.08	3.33	3.08 to 3.59	3.74^a^	3.40 to 4.09
Conditional length at 24 mo	1.45	1.29 to 1.61	1.51	0.67 to 2.34	1.34	1.09 to 1.59	1.30	0.94 to 1.66
Conditional length in mid-childhood	1.93	1.76 to 2.09	3.27	2.42 to 4.11	1.92	1.67 to 2.16	2.23	1.88 to 2.57
Systolic blood pressure, mm Hg								
Birth weight	−0.44^a^	−1.06 to 0.17	−7.49^b^	−13.72 to −1.25	0.90^a^	−0.41 to 2.22	0.38^a^	−0.49 to 1.24
Conditional length at 12 mo	0.65^b^	0.21 to 1.08	1.31	−1.39 to 4.01	0.71	0.02 to 1.40	1.70^a^	0.82 to 2.58
Conditional length at 24 mo	0.73	0.29 to 1.16	2.02	−0.21 to 4.25	0.66	−0.03 to 1.35	1.11	0.19 to 2.03
Conditional length in mid-childhood	0.59	0.15 to 1.03	1.04	−1.20 to 3.27	0.57	−0.08 to 1.22	0.94	0.05 to 1.83
Diastolic blood pressure, mm Hg								
Birth weight	−0.52^b^	−1.05 to 0.01	−1.99	−7.47 to 3.50	0.74^a^	−0.42 to 1.90	−0.18	−0.91 to 0.54
Conditional length at 12 mo	0.48	0.11 to 0.86	0.58	−1.79 to 2.96	0.64	0.03 to 1.25	1.15	0.41 to 1.89
Conditional length at 24 mo	0.35	−0.02 to 0.72	1.48	−0.48 to 3.44	0.53	−0.07 to 1.14	0.31	−0.47 to 1.08
Conditional length in mid-childhood	0.17	−0.21 to 0.55	0.13	−1.84 to 2.10	0.48	−0.10 to 1.05	0.18	−0.57 to 0.93
Fasting glucose, mmol/L								
Birth weight	−0.04	−0.08 to 0.00	−0.09	−0.48 to 0.29	0.02	−0.08 to 0.12	−0.07	−0.13 to 0.00
Conditional length at 12 mo	0.01	−0.02 to 0.04	0.03	−0.15 to 0.20	0.01	−0.05 to 0.06	−0.03	−0.10 to 0.04
Conditional length at 24 mo	0.02	−0.01 to 0.05	0.01	−0.14 to 0.15	0.01	−0.04 to 0.06	0.02	−0.05 to 0.10
Conditional length in mid-childhood	0.01	−0.02 to 0.04	0.08	−0.07 to 0.22	0.00	−0.05 to 0.05	−0.01	−0.08 to 0.06
Completed years of schooling								
Birth weight	0.20^a^	0.05 to 0.34	0.41	−1.21 to 2.03	0.63^b^	0.28 to 0.97	0.03^a^	−0.22 to 0.29
Conditional length at 12 mo	0.57	0.46 to 0.67	−0.06^b^	−0.76 to 0.64	0.70	0.52 to 0.89	0.75^a^	0.49 to 1.01
Conditional length at 24 mo	0.51	0.41 to 0.61	0.38	−0.20 to 0.96	0.38	0.20 to 0.56	0.57	0.30 to 0.84
Conditional length in mid-childhood	0.23	0.13 to 0.34	0.21	−0.38 to 0.79	0.20	0.02 to 0.37	0.26	0.00 to 0.52
Prehypertension/hypertension, OR								
Birth weight	0.97	0.85 to 1.10	0.58	0.16 to 2.10	1.25	0.97 to 1.62	0.93	0.76 to 1.13
Conditional length at 12 mo	1.13	1.03 to 1.24	1.13	0.64 to 1.99	1.04	0.91 to 1.19	1.24	1.01 to 1.52
Conditional length at 24 mo	1.07	0.98 to 1.18	1.19	0.74 to 1.92	1.11	0.97 to 1.27	1.01	0.82 to 1.25
Conditional length in mid-childhood	1.04	0.95 to 1.14	1.02	0.64 to 1.63	1.16	1.02 to 1.32	1.10	0.90 to 1.35
IFG/diabetes, OR								
Birth weight	0.98	0.74 to 1.29	(-)§		0.84	0.56 to 1.26	0.65	0.44 to 0.96
Conditional length at 12 mo	0.95	0.78 to 1.16			0.83	0.66 to 1.05	0.70	0.47 to 1.04
Conditional length at 24 mo	1.37	1.12 to 1.67			0.89	0.71 to 1.12	1.14	0.77 to 1.68
Conditional length in mid-childhood	0.96	0.79 to 1.17			1.15	0.92 to 1.44	0.92	0.65 to 1.31
Completed secondary school, OR¶								
Birth weight	1.28	1.11 to 1.48	1.28	0.32 to 5.04	1.52	1.12 to 2.06	1.11	0.91 to 1.36
Conditional length at 12 mo	1.41	1.27 to 1.56	1.33	0.73 to 2.40	1.45	1.23 to 1.72	1.46	1.18 to 1.81
Conditional length at 24 mo	1.37	1.24 to 1.52	1.36	0.82 to 2.24	1.37	1.17 to 1.62	1.27	1.02 to 1.58
Conditional length in mid-childhood	1.15	1.04 to 1.28	1.29	0.80 to 2.09	1.12	0.97 to 1.31	1.23	1.01 to 1.51

Data are coefficients and associated 95% CIs from models in which the adult outcome is predicted from the childhood conditional size measures. Separate regression models were developed for each adult outcome within each birth category. For continuous measures, coefficients represent the change in the adult variable associated with a 1 SD change in the conditional size measure. For binary measures, the coefficients represent the OR per 1 SD change in the conditional size measure. Models are adjusted for site, sex, and age at adult measurement. Within any row, differing superscript letters denote P < .05 by the Tukey least significant difference test.

∗ LGA: above the 90th percentile of sex-specific distribution of weight for GA.

† SGA: below the 10th percentile of sex-specific distribution of weight for GA.

‡ Preterm: <37 completed weeks gestation, based on date of LMP.

§ Only 3 participants who were Term-LGA had prediabetes/diabetes. The model fit for the preterm category is questionable owing to limited sample size.

¶ Completion of secondary school: 12th grade in Brazil, India, and South Africa; 11th grade in Guatemala; and 10th grade in Philippines. The model fit for the not preterm- LGA category is questionable owing to limited sample size.

## Appendix

The Consortium of Health-Orientated Research in Transitioning Societies group members include: Pelotas Birth Cohort (Brazil): Fernando C. Barros, Denise Gigante, Pedro C. Hallal, Bernardo L. Horta, Cesar G. Victora (Universidade Federal de Pelotas, Pelotas). Institute of Nutrition of Central America and Panama Nutrition Trial Cohort Study (Guatemala): Yaw O. Addo, Wei Hao, Reynaldo Martorell, Aryeh D. Stein (Emory University, Atlanta GA); Manual Ramirez-Zea (Instituto de Nutrición de Centra América y Panamá, Guatemala). New Delhi Birth Cohort Study (India): Harshpal S. Sachdev, Shikha Sinha (Sitaram Bhartia Institute for Science and Research, New Delhi); Lakshmi Ramakrishnan, Nikhil Tandon (All-India Institute of Medical Sciences, New Delhi); Dorairaj Prabhakaran (Centre for the Control of Chronic Diseases, New Delhi); K. Srinath Reddy (Public Health Foundation of India, New Delhi); Anita Khalil (The Heart Centre, New Delhi); Poornima Prabhakaran (Initiative for Cardiovascular Health Research in Developing Countries, New Delhi); S. K. Dey Biswas (Indian Council of Medical Research, New Delhi); Santosh Bhargava, Tarun Gera, K. D. Gupta, Arti Mishra, Dinesh Mishra, Bhaskar Singh, Rajeshwari Verma (S.L. Jain Hospital, Delhi); Siddarth Ramji (Maulana Azad Medical College, New Delhi); Caroline H. D. Fall, Clive Osmond (University of Southampton, Southampton, UK). Cebu Longitudinal Health and Nutrition Study (Philippines): Linda S. Adair (University of North Carolina at Chapel Hill, Chapel Hill, NC); Isabelita Bas, Judith Borja, Nanette Lee (Office of Population Studies, University of San Carlos, Cebu City); Darren L. Dahly (University of Leeds, Leeds); Christopher W. Kuzawa (Northwestern University, Evanston IL). Birth to Twenty (South Africa): Julia De Kadt, Shane A. Norris Linda Richter, (University of the Witwatersrand, Johannesburg); Alan Stein (University of Oxford, Oxford).
